# Tea Polyphenols Protect the Mammary Gland of Dairy Cows by Enhancing Antioxidant Capacity and Regulating the TGF-β1/p38/JNK Pathway

**DOI:** 10.3390/metabo12111009

**Published:** 2022-10-22

**Authors:** Ran Xu, Mengran Zhu, Jingwen Cao, Mengyao Guo

**Affiliations:** College of Veterinary Medicine, Northeast Agricultural University, Harbin 150030, China

**Keywords:** mammary gland, transcriptomic analysis, tea polyphenols, antioxidant capacity, inflammation, TGF-β1 pathway

## Abstract

Tea polyphenols (TPs) are the main active substances in tea and they have many beneficial effects, such as anti-inflammation, antioxidant, anti-cancer and metabolic regulation effects. The quality of milk is affected by mammary gland diseases and there are substantial economic losses resulting from reduced milk production as a consequence of inflammatory injury of the mammary gland. In this study, transcriptome analysis and molecular biology techniques were used to study the effects of TPs on inflammatory injury of the mammary gland. After intervention with TPs, a total of 2085 differentially expressed genes were identified, including 1189 up-regulated genes and 896 down-regulated genes. GO analysis showed that differentially expressed genes played an important role in proton transmembrane transport, oxidation–reduction reactions and inflammatory response. KEGG enrichment suggested that differential genes were concentrated in the TGF-β pathway and active oxygen metabolism process. Experiments were performed to confirm that TPs increased SOD, CAT, T-AOC and GSH-Px content along with a reduction in MDA. Meanwhile, TPs inhibited the expression of TGF-β1 and reduced the phosphorylation of p38 and JNK. The expression of inflammatory cytokines IL-1β, IL-6 and TNF-α were significantly decreased after intervention with TPs. In summary, all the data indicated that TPs protected the mammary gland by enhancing the antioxidant capacity and down-regulating the TGF-β1/p38/JNK pathway.

## 1. Introduction

Mammary gland diseases have a great impact on the dairy product industry, not only resulting in a sharp drop in milk production but also greatly increasing the product safety risks [1]. Inflammatory injury of the mammary gland is one of the main diseases of the mammary gland in dairy cows. It is characterized by the infection of the mammary gland, which is accompanied by an increase in temperature, swelling of the mammary gland, redness and pain, a change in the characteristics of the milk and other symptoms [2]. Inflammatory injury of the mammary gland increases the permeability of the blood–breast barrier, causing a decrease in milk fat, carbohydrate and energy levels [3]. This degrades the quality of the milk. Furthermore, inflammatory injury of the mammary gland reduces a cow’s fertility and affects reproduction [4]. Therefore, protecting mammary gland health has become a significant problem to be solved in dairy farms.

Related studies have shown that milk yield is closely related to the state of the mitochondria [5]. Mitochondria, also referred to as “power houses”, are the main sites of aerobic respiration in cells. During the synthesis of ATP, an electrochemical gradient is established and oxygen is consumed [6,7]. In normal physiological conditions, reactive oxygen species (ROS) produced by mitochondria are in a dynamic balance [8]; however, once the oxidation–reduction reactions are unbalanced, the accumulation of ROS occurs [9]. The outcome of the rivalry between ROS and antioxidants determines whether the body is in a state of oxidative stress. Excessive accumulation of ROS disrupts cell homeostasis and causes mitochondrial dysfunction, leading to oxidative stress [10]. ROS are pivotal signaling molecules that contribute to the progression of inflammatory diseases. An excess of ROS triggers an inflammatory response, which is one of the important factors contributing to inflammatory injury of the mammary gland [11,12]. NF-kB, IL-6/JAK/STAT3, p38 MAPK and PI3K are well-known, classical pathways of inflammatory expression [13,14]. It has been documented that inflammatory injury of the mammary gland is inhibited by the down-regulation of the NF-kB and MAPK signaling pathways [15]. However, it remains unclear whether the mammary gland is affected by additional inflammatory signaling pathways. Besides antibiotics, no other drugs have been found to have a good effect on the inflammatory injury, while the negative effects of antibiotic resistance compel us to find alternatives.

In recent years, natural foods have received increasing attention because of the important role they play in disease prevention and treatment. Tea is a beverage with a long history, and different fermentation times determine the various types of tea [16]. Although the types of tea vary, they all have active ingredients called tea polyphenols (TPs). TPs are the bioactive ingredients of tea that can prevent many diseases, such as cancer, stroke and arthritis [17]. Flavonoids are the main components of polyphenols, accounting for about 60%, while phenolic acids and other polyphenols account for about 40% [18]. As natural compounds present in tea, TPs have good anti-inflammatory and antioxidant properties [19,20,21]. However, the effect of TPs on the bovine mammary gland is still unclear. Thus, transcriptome analysis and molecular biology techniques were used to determine the mechanism of the effect of TPs on mammary gland.

## 2. Materials and Methods

### 2.1. Experimental Animal Treatment

A total of 20 Holstein cows, aged 7 years with a similar lactation period and three births were provided by the Northeast Agricultural University Laboratory Animal Research Center (Harbin, China). The cows were equally divided into two groups. The experimental protocol of this study was in accordance with animal welfare regulations and approved by the Animal Care and Use Committee of Northeast Agricultural University. The modeling method used for the TPs (40.83% epigallocatechin gallate (EGCG), 12.99% epigallocatechin (EGC), 9.28% epicatechin gallate (ECG) and 5.20% epicatechin (EC)) treatment group (TG) was as follows: milk catheters of cows were perfused with TPs at a dose of 6 mg/kg every two days, five times in total. The dose of TPs was determined based on the significant reduction in ROS expression in the pre-experiment. The modeling method for the control group (CG) was to inject normal saline into milk ducts at the same frequency as the TG group. During modeling, the cows were kept in a temperature-controlled environment and provided with adequate water and feed. After modeling, procaine was used for local anesthesia, a puncture needle was inserted into the breast, and the mammary gland tissue was collected by gentle suction with a micro-sampler. Samples were stored in a refrigerator at −80 °C.

### 2.2. Transcriptomic Analysis

#### 2.2.1. RNA Extraction

Appropriate amounts of the mammary gland were taken from the CG and TG, and Trizol (Invitrogen Life Technology Co., LTD, Carlsbad, CA, USA) was used to lyse the tissues and cells on ice. Chloroform, isopropyl alcohol and DEPC alcohol were added for purification. We dissolved the RNA in DEPC water, and total RNA was extracted. The cDNA library was further established, and the quality and sequencing of the library were detected.

#### 2.2.2. RNA Sequencing and Quality Control

After sequencing, the original data in FASTQ format was converted from the original image file BCL after base recognition, and its quality was analyzed. Sequencing quality and base composition analysis were the main purposes of the quality analysis. Low-quality sequences were removed based on the results of the quality analysis, and the quality of cleaning data was evaluated using FASTQC V0.10.1.

#### 2.2.3. Differential Expression Analysis

EBSeq software was used for the determination of differential expressions in the data. We drew a volcano plot according to the differential expression of different genes. When FDR < 0.05 and |log_2_FC| > 1, this indicated that there were significant differences in the expression of transcripts and genes in biological replication. Gene Ontology (GO) functional enrichment analysis was mainly carried out by the GOseq R package [22]. When Q < 0.05, it was considered that the differentially expressed genes (DEGs) were significantly enriched. The DEGs enriched in the Kyoto Encyclopedia of Genes and Genomes (KEGG) biological pathway enrichment were analyzed by KOBAS (V3.0) software.

### 2.3. Determination of Reactive Oxygen Species (ROS) Expression

ROS expression was determined by a ROS kit (Jiancheng Bioengineering Institute, Nanjing, China). A single-cell suspension was prepared from the mammary gland samples. The cell precipitates were suspended by diluted DCFH-DA in sample tubes, which were incubated at 37 °C for 60 min. The excitation wavelength was set at 500 nm and the emission wavelength was set at 525 nm for fluorescence detection. The corresponding fluorescence values were read.

### 2.4. MAC-T Cell Culture

Bovine mammary epithelial cell line MAC-T was cultured in DME/F-12 medium added with 100 U/mL penicillin /100 μg/mL streptomycin (Invitrogen Life Technology Co., LTD, Carlsbad, CA, USA) and 10% fetal bovine serum (Thermo Fisher Scientific, Waltham, MA, USA). The cells were placed in an incubator at 37 °C and 5% CO_2_. The cells were placed on a cell plate and divided into five groups: CG, H_2_O_2_, LTG, NTG and HTG. CG was the control group, the cells were routinely cultured. H_2_O_2_ was the hydrogen peroxide stimulation group. LTG was the H_2_O_2_ + 20 μg/mL TPs treatment group. NTG was the H_2_O_2_ + 40 μg/mL TPs treatment group. HTG was the H_2_O_2_ + 60 μg/mL TPs treatment group. The supernatant of each group was collected at the same time.

### 2.5. Immunofluorescence

MAC-T cells grew on the slides when they were immersed in a cell culture medium. The slides were fixed with 4% paraformaldehyde for 15 min, and then they were immersed in PBS. They were permeated with 0.5% Triton X-100 at room temperature for 20 min. Normal goat serum was added to the slides, and the slides were sealed at room temperature for 30 min. A sufficient amount of diluted primary antibody (Cytokeratin 18) was dropped onto each slide, which was then placed in a wet box for overnight incubation at 4 °C. After washing, the diluted fluorescent secondary antibody was dropped onto the slide and the slides were incubated in a wet box at 37 °C for 1 h. DAPI was dropped onto the slides and they were incubated for 5 min to avoid light. The slides were sealed with a product containing an anti-fluorescence quench agent, and then we observed and collected the images with a fluorescence microscope.

### 2.6. Cell Viability Assay

MAC-T cell suspension was inoculated on 96-well plates and the cell density was controlled at 5000 cells per well. The concentration of TPs was as follows: 0, 10, 20, 30, 40, 50, 60, 70, 80, 90 and 100 μg/mL. TPs were added to the 96-well plate and treated for 12 h. CCK-8 solution was added to each well. The cells with CCK-8 were incubated in the cell incubator for 1 h, then we measured the OD value at 450 nm with a microplate reader.

### 2.7. Measurement of Oxidative and Antioxidant Capacity

To identify the related oxidation and antioxidant capacity of the mammary gland and MAC-T cell, MDA, CAT, SOD and T-AOC were determined by using the related detection kits (Jiancheng Bioengineering Institute, Nanjing, China). MDA is one of the products of lipid peroxides, so the amount of MDA reflects the degree of lipid peroxidation in the body, and indirectly reflects the degree of cell damage. SOD can remove superoxide anion free radicals, CAT can decompose hydroxyl free radical, GSH-Px can catalyze the decomposition of hydrogen peroxide, and T-AOC can measure the total antioxidant capacity of both enzymatic and non-enzymatic systems. Therefore, the organism protects against oxidation by eliminating free radicals and reactive oxygen species to prevent lipid peroxidation, breaking down peroxides, blocking peroxidation chains, and removing catalytic metal ions. The detection of the corresponding indexes reflected the antioxidant capacity of the organism. All measurements were carried out according to the kit manufacturer’s instructions.

### 2.8. Real-Time qPCR

RNA was extracted from mammary gland tissue and MAC-T cells. The tissues were frozen with liquid nitrogen and fully ground, and the cells were washed with PBS. Appropriate amounts of tissues and cells were fully lysed on ice with Trizol. A mixture of chloroform, isopropyl alcohol and 75% ethanol was extracted and purified by centrifugation. The RNA was reverse transcribed into cDNA using a reverse transcription kit. Primer Premier (v5.00) software was used to design Primer sequences as follows. Glyceraldehyde 3-phosphate dehydrogenase (GAPDH) was used as the internal reference gene, and qPCR was performed using the SYBR Green Plus reagent kit and fluorescence quantitative instrument according to the instructions. The mRNA relative abundance of each gene was calculated by the 2^−ΔΔCt^ method.

### 2.9. ELISA

The expression of TGF-β1 and inflammatory factors IL-1β, IL-6 and TNF-α were detected by ELISA kits (Jiancheng Bioengineering Institute, Nanjing, China). An appropriate amount of mammary gland was taken, 1 mL phosphate buffer saline was fully ground with the tissue using a homogenizer, and the supernatant was collected after centrifugation at 4 °C at 2000 rpm for 40 min. MAC-T cells were cultured and starved without serum DMEM/F-12. The supernatant was taken for an enzyme-linked immunosorbent assay. TGF-β1, IL-1β, IL-6 and TNF-α were detected by ELISA kits according to the instructions, and the OD value was obtained at 450 nm within 15 min. At the same time, the mean standard deviation of all independent experiments was used to express the results.

### 2.10. Measurement of Inflammation-Related Proteins Phosphorylation

Phosphorylation of TGF-β receptor Ⅰ, Ⅱ and related pathway proteins p38 and JNK were detected by ELISA kits (Cell Signaling Technology, Danvers, MA, USA). According to the manufacturer’s instructions, we diluted the solution and reagent in pure water. Cell lysates of tissues and cells were extracted, removed and added to the appropriate wells. Micro-holes were sealed with tape and firmly pressed. The plates were incubated at 37 °C for 2 h. After washing, detection antibodies were added and HRP-labeled secondary antibodies were added for incubation and washing. We repeated the washing process, added TMB substrate, sealed the plates with tape and incubated them. The absorbance at 450 nm was read within 30 min after the addition of the stop solution.

### 2.11. Statistical Analysis

GraphPad Prism version 8.0.1 software was used to perform the statistical analyses. In the in vivo experiments, the T test was performed with tea polyphenols as variables, the H_2_O_2_ group was used as a positive control in the in vitro experiments, and tea polyphenols were used as variables for one-way ANOVA. Results of three parallel experiments were represented as the mean and standard deviation (M ± SD), and compared to the control group. Samples with an asterisk (*) show a significant difference (*p* < 0.05).

## 3. Results

### 3.1. Analysis of DEGs in Mammary Gland after Intervention with TPs

**Figure 1:**
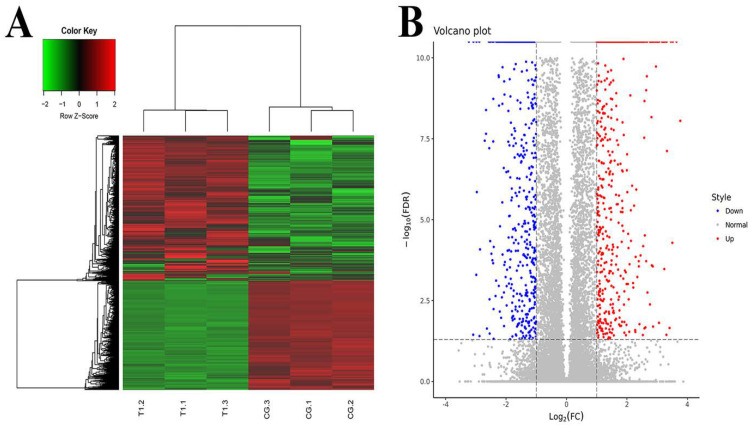
Differentially expressed genes analysis. (**A**) Clustering of differentially expressed genes. Hierarchical clustering is based on FPKMs, where log_10_ (FPKM + 1) is used for clustering. The red color represents genes with higher expression, while the green color represents genes with lower expression. (**B**) Volcano plot of differential expression gene. Red represents genes with higher expression; blue represents genes with lower expression. CG, control group, milk duct perfusion with 6 mg/kg normal saline. T, tea polyphenols group, milk duct perfusion 6 mg/kg tea polyphenols.

The DEGs were screened by EBSeq. The number of DEGs in the TG and CG was as follows. A total of 62,658 genes were detected, and 2085 genes were differentially expressed, of which 1189 genes were significantly up-regulated and 896 genes were significantly down-regulated. RPKM/COUNT was used as the expression level of differential genes. Hierarchical clustering analysis was adopted as shown in Figure 1A. Regions with different colors were used to represent different cluster grouping information, and genes with the same or similar expression patterns were clustered to determine the clustering patterns of different sample control patterns. In the volcano plot, different colors were used to represent different gene expressions, and up-regulated genes and down-regulated genes were placed on both sides of genes with no significant difference in expression (Figure 1B).

### 3.2. Analysis of GO Enrichment in Mammary Gland after Intervention with TPs

**Figure 2:**
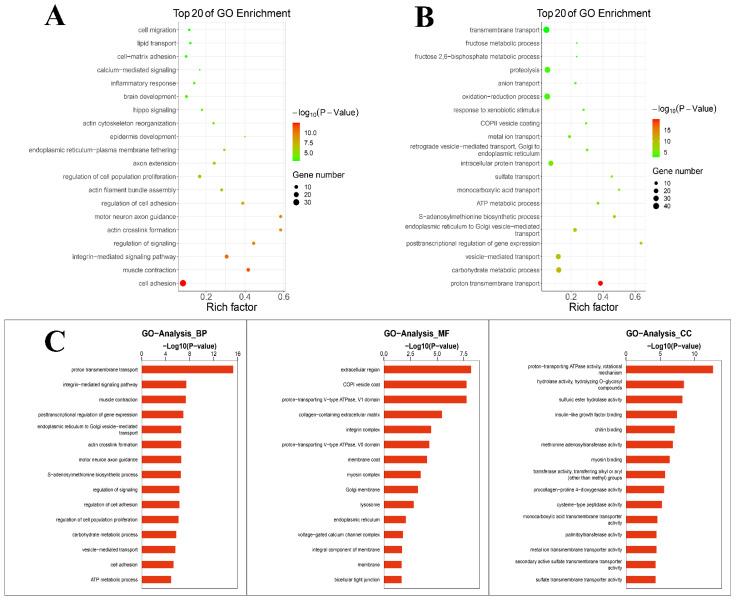
GO enrichment analysis. (**A**) GO enrichment analysis of down-regulated genes. (**B**) GO enrichment analysis of up-regulated genes. (**C**) A summary of the biological processes, molecular functions and cellular components of differentially expressed genes.

The cellular components, molecular functions and biological processes of the DEGs were explored through GO enrichment analysis. GO enrichment analysis was performed for up-regulated and down-regulated genes. The results (Figure 2A) showed that DEGs mainly down-regulated the inflammatory response, etc. The results (Figure 2B) showed that different genes mainly up-regulated the oxidation–reduction process, etc. Figure 2C shows a summary of the biological process, cellular components and molecular functions of DEGs. DEGs enriched in terms of the biological process were mainly distributed in transport and biological processes. Within the scope of molecular function, DEGs were distributed in the transport function. With regard to the cellular component, DEGs were mostly distributed in enzyme activity. GO enrichment analysis suggested that TPs affected the physiological process during lactation, balanced oxidation–reduction and reduced the inflammatory response.

### 3.3. The KEGG Pathway Enrichment Analysis of DEGs

**Figure 3:**
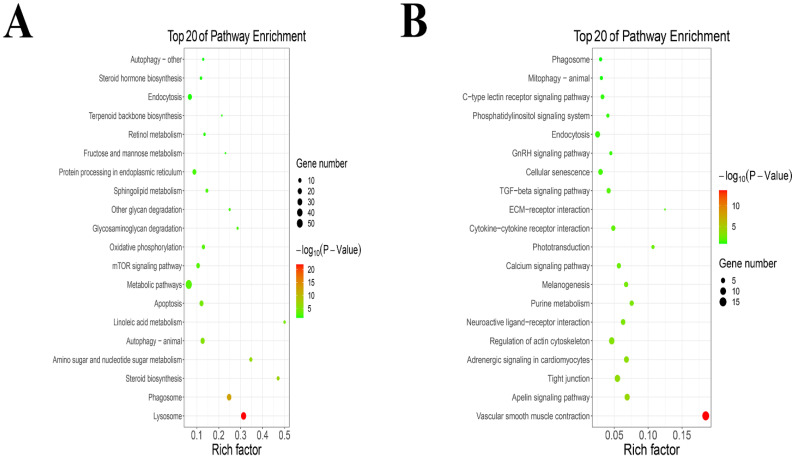
The KEGG pathway enrichment analysis. (**A**) The KEGG pathway enrichment analysis of up-regulated genes. (**B**) The KEGG pathway enrichment analysis of down-regulated genes.

The KEGG pathway analysis results are shown in the KEGG pathway bubble diagram in Figure 3. KEGG enrichment results show that TPs increased the expression of lysosome, phagosome, steroid biosynthesis, amino sugar and nucleotide sugar metabolism, autophagy (animal), linoleic acid metabolism, apoptosis, metabolic pathways, mTOR signaling pathway, oxidative phosphorylation, glycosaminoglycan degradation, other glycan degradation, sphingolipid metabolism, protein processing in endoplasmic reticulum, fructose and mannose metabolism, retinol metabolism, terpenoid backbone biosynthesis, endocytosis, steroid hormone biosynthesis and autophagy (other) (Figure 3A). An addition, TPs decreased the expression of vascular smooth muscle contraction, apelin signaling pathway, tight junction, adrenergic signaling in cardiomyocytes, regulation of actin cytoskeleton, neuroactive ligand−receptor interaction, purine metabolism, melanogenesis, calcium signaling pathway, phototransduction, cytokine−cytokine receptor interaction, ECM−receptor interaction, TGF−beta signaling pathway, cellular senescence, GnRH signaling pathway, endocytosis, phosphatidylinositol signaling system, C−type lectin receptor signaling pathway, mitophagy of animal and phagosome (Figure 3B).

### 3.4. Effects of TPs on Antioxidant Capacity and Expression of Inflammatory Factors

**Figure 4:**
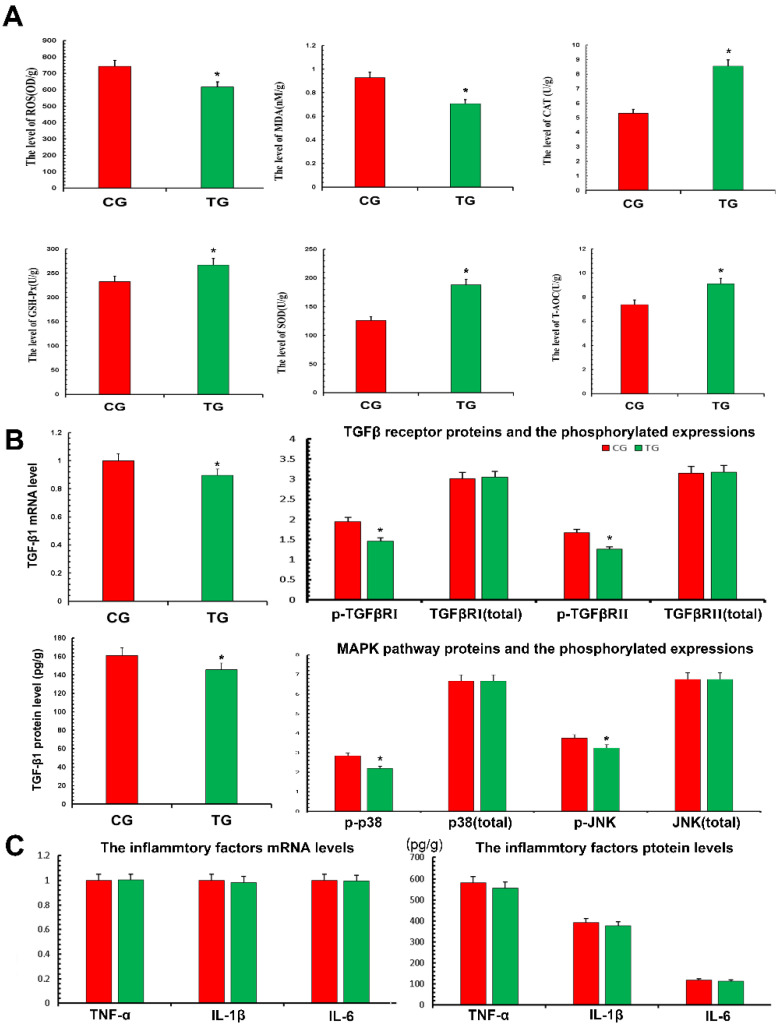
Antioxidant capacity and inflammatory factors expressed in the mammary gland. Proteins expressions were detected with ELISA kits. RT-qPCR was used to verify mRNA expression. GAPDH was used as a control. (**A**) The measurement of oxidative damage and antioxidant capacity. (**B**) The detection of TGF-β-associated inflammatory signaling pathway. (**C**) The proteins expressions of TNF-α, IL-1β and IL-6. CG, control group, milk duct perfusion with 6 mg/kg normal saline. TG, tea polyphenols group, milk duct perfusion 6 mg/kg tea polyphenols. The asterisk represents the comparison with CG, and *p* < 0.05 is significantly different from CG. The experimental data represent the mean ± SD of three independent experiments.

In terms of oxidation and antioxidant capacity, the expression of ROS and MDA in the TG was significantly decreased compared with the CG, while the expression of CAT, SOD, GSH-Px and T-AOC was significantly increased. This demonstrated that TPs reduced oxidative stress and improved antioxidant capacity (Figure 4A). According to the results of transcriptomics, the expression of the TGF-β pathway decreased, thus the relevant TGF-β1, TGF-β receptors and phosphorylation of p38 and JNK were detected (Figure 4B). The results showed that the expression of TGF-β1 in the TG decreased significantly compared with the CG. The results also showed that the phosphorylation levels of TGF-β receptor Ⅰ, Ⅱ and related inflammatory pathway proteins decreased after treatment with TPs. This suggested that TPs inhibited the expression of inflammation. This study used RT-qPCR and ELISA to detect the expression of inflammatory factors affected by TP. As shown in Figure 4C, mRNA and protein expressions of TNF-α, IL-1β and IL-6 in the TG decreased slightly compared with the CG, but the decrease was not significant. The reason was presumed to be that CG and TG were not in a disease state, and therefore, inflammatory factors were not expressed significantly.

### 3.5. Cell Identification and Effect of TPs on Cell Viability

**Figure 5:**
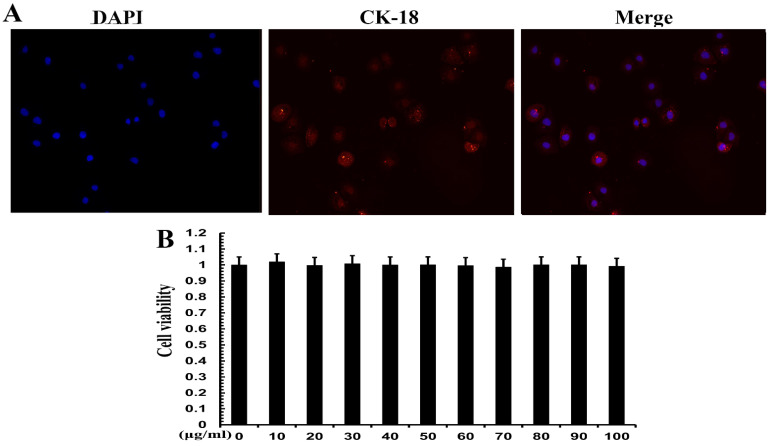
Cell identification and viability analysis of MAC-T cells. (**A**) Cell electron microscopy image. Blue represents the nucleus and red represents the expression of cytokeratin 18 (CK-18). (**B**) Cell viability at different concentrations. The numbers 0–100 indicate the concentration of tea polyphenols in μg/mL.

Positive expression of CK-18 is evidence of an epithelial lineage. CK-18 immunofluorescence was used for cell identification. The results showed that CK-18 was highly expressed in MAC-T cells (Figure 5A), which proved that MAC-T cells were mammary epithelial cells. CCK-8 assay was used to verify the cell viability of TPs. The results show that 20, 40 and 60 mg/mL of TPs had no significant effect on cell survival. TPs at 10, 30, 50, 70, 80, 90 and 100 mg/mL had a slight effect on cell survival but the difference was not significant (Figure 5B). Therefore, it could be inferred that TPs in this range of doses had no cytotoxicity.

### 3.6. TPs Reduced Inflammatory Damage by Enhancing Antioxidant Capacity and Inhibiting the TGF-β1/p38/JNK Pathway

**Figure 6:**
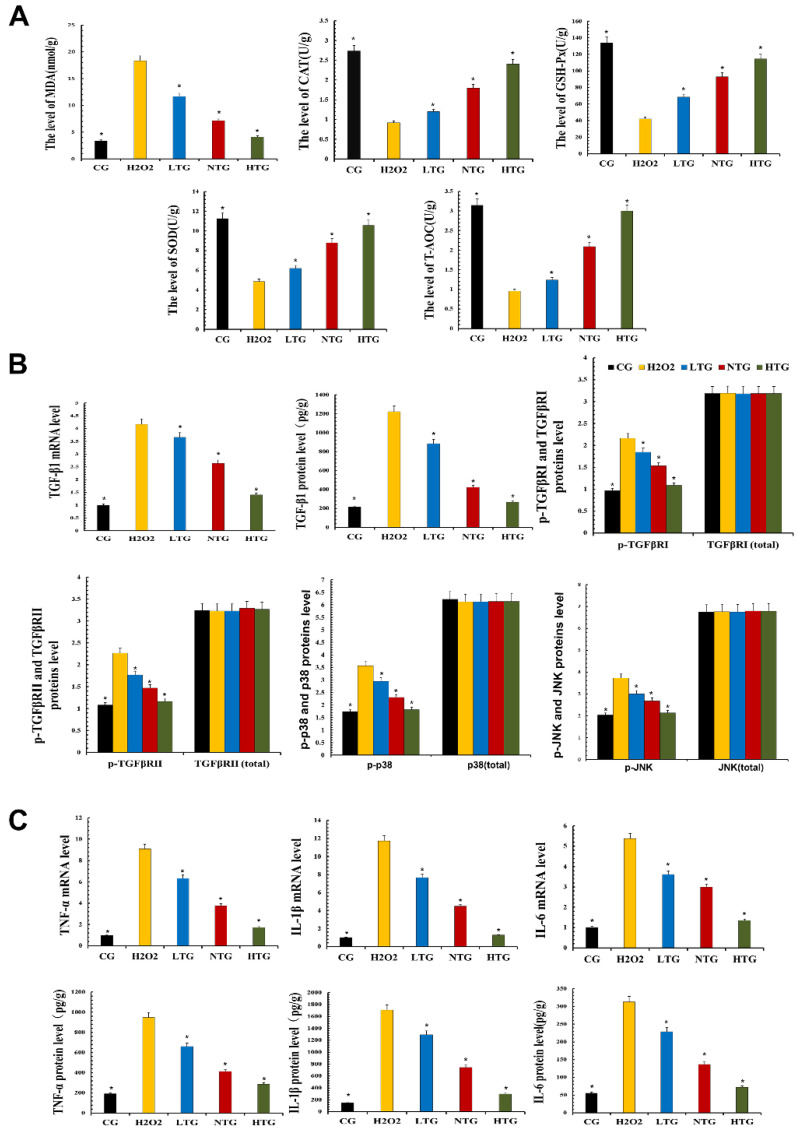
Oxidative stress and inflammatory response in MAC-T cells. Proteins’ expressions were detected with ELISA kits. RT-qPCR was used to verify mRNA expression. GAPDH was used as a control. (**A**) The measurement of oxidative damage and antioxidant capacity. (**B**) The expression of TGF-β1 mRNA and protein and the expression of proteins of the TGF-β signaling inflammatory signaling pathway. (**C**) The mRNA and protein expressions of inflammatory cytokines. CG, control group, the cells were routinely cultured. H_2_O_2_, hydrogen peroxide stimulation group. LTG, H_2_O_2_ + 20 μg/mL tea polyphenols treatment group. NTG, H_2_O_2_ + 40 μg/mL tea polyphenols treatment group. HTG, H_2_O_2_ + 60 μg/mL tea polyphenols treatment group. The asterisk represents the comparison with H_2_O_2_, and *p* < 0.05 is significantly different from H_2_O_2_. The experimental data represent the mean ± SD of three independent experiments.

To test the antioxidant capacity of TPs, MAC-T cells treated with TPs were tested with related kits. The experimental results revealed that the expression levels of SOD, CAT, GSH-Px and T-AOC in LTG, NTG and HTG were significantly increased in the presence of TPs compared with H_2_O_2_. At the same time, the expression of antioxidant enzymes increased with the increase in TPs content and the expression of MDA in LTG, NTG and HTG decreased significantly, indicating the intervention of TPs-reduced oxidative stress (Figure 6A).

According to the transcriptomics results, the expression of the TGF-β pathway was decreased. Verification was performed on MAC-T cells, and the expression of TGF-β1, the phosphorylation levels of TGF-β receptor Ⅰ, receptor Ⅱ, p38 and JNK were detected. Related inflammatory pathway proteins were decreased after TPs treatment, suggesting that TPs inhibited phosphorylation of TGF-β receptors in the inflammatory pathway and decrease inflammatory expression (Figure 6B).

RT-qPCR and ELISA kits were used to detect the expression of inflammatory factors at the gene and protein levels. Compared with the CG, RT-qPCR and ELISA results showed that the expression of TNF-α, IL-1β and IL-6 were significantly increased in the H_2_O_2_ group. After the addition of TPs, the experimental data showed that the expressions of inflammatory factors decreased significantly. The effect of TPs was dose-dependent (Figure 6C). This also demonstrated the anti-inflammatory effect of TPs.

## 4. Discussion

Mammary gland diseases affect the quality of milk and even cause the complete loss of the lactation function of dairy cows. The decline in milk quality and yield has resulted in serious economic losses [23]. Among them, the inflammatory injury of the mammary gland is an important factor that affects the development of dairy farming. With the increase in age and parity of cows, the probability of inflammatory injury of the mammary gland has gradually increased. Milk accumulation and nipple rupture are the main causes of infectious mammary gland diseases in dairy cows [24,25]. However, with the development of dairy modernization, the inducement of inflammatory injury in mammary glands also changes. Relevant studies have shown that oxidative stress induced by negative energy balance, production stress and other factors can lead to mammary gland damage in dairy cows [26,27]. Oxidative stress is caused when the dynamic balance of the oxidation–reduction reaction is broken [28]. If the adverse factors causing oxidative stress cannot be eliminated, inflammation will be further induced [29]. Studies have shown that some natural compounds effectively reduce the damage to the mammary gland and the occurrence of diseases, and play a protective role. TPs are the main active substances in tea and have many benefits, in particular, their excellent antioxidant and anti-inflammatory abilities [30,31]. In our study, we found that TPs significantly enhanced the antioxidant capacity of mammary glands.

Transcriptomics has been used extensively in conducting bioactivity assessments. In the present study, transcriptomic results showed that TPs could regulate the expression of mammary gland genes in dairy cows, and 2085 DEGs were screened. Further analysis confirmed that TPs up-regulated the antioxidant capacity of the mammary gland and decreased the inflammatory response. A large number of studies have confirmed that antioxidant capacity is closely related to the occurrence of inflammation. The improvement of antioxidant capacity can play a protective role in tissues by regulating a variety of signaling pathways. For example, increased antioxidant capacity can protect the liver from non-alcoholic fatty liver disease [32], protect the colon and reduce the inflammatory response [33]. Therefore, according to the results of transcriptome analysis, tea polyphenols should have a protective effect on regulating the antioxidant capacity of the mammary gland and reducing the inflammatory injury.

The protective effect of TPs on the mammary gland was further confirmed and its mechanism was explored. The results revealed that TPs significantly increased the activity of antioxidant enzymes and reduced the production of peroxide products and ROS. ROS is a by-product of cellular aerobic respiration [34,35], and MDA is an important marker of lipid peroxidation [36]. CAT, SOD, GSH-Px and T-AOC are significant indexes for testing antioxidant capacity and the level of their expression determine the level of antioxidant capacity [37]. However, the signaling pathways by which ROS-induced secretion of inflammatory factors caused mammary gland damage are still unclear. Transcriptome analysis showed that TGF-β pathway was closely related to TPs. TGF-β is an effective immunosuppressive factor associated with inflammation, autoimmune and cancer [38]. TGF-β receptors Ⅰ and Ⅱ have kinase activity that activates regulated or non-standard signaling pathways and modulates the activation of other pathways [39]. Studies have shown that TGF-β can activate the MAPK signaling pathway and induce inflammatory injury. JNK and p38 MAPK, the classic pathways of inflammatory signaling [40] are transmitted through phosphorylation of p38 and JNK proteins [41]. The phosphorylation of p38 and JNK promotes the secretion of inflammatory factors and initiates the cell death program. Studies have shown that TGF-β1 expression increases the risk of cardiovascular inflammation in the adult population [42], and the inhibition can alleviate cellular inflammatory expression and play a protective role [43]. The results of this experiment showed that phosphorylation levels of p38 and JNK were significantly reduced under TPs treatment, suggesting that TPs protect the mammary gland and inhibit the inflammatory response pathway.

## 5. Conclusions

In the present study, TPs reduced the excessive accumulation of ROS by regulating gene expression in mammary tissue of dairy cows and enhancing the activity of antioxidant enzymes. Meanwhile, TP inhibited the production of ROS-induced inflammatory factors by reducing the expression of TGF-β1 and its receptors, as well as the phosphorylation of p38 and JNK. This study provides a new option for protecting mammary gland health and preventing mammary gland damage in dairy cows.

## Data Availability

Not applicable.
